# The effect of co supplementation of omega-3 and vitamin D on cardio metabolic risk factors and psychological distress in reproductive-aged women with prediabetes and hypovitaminosis D: a study protocol for a randomized controlled trial

**DOI:** 10.1186/s13063-019-3948-5

**Published:** 2019-12-30

**Authors:** Masoumeh Rajabi-Naeeni, Mahrokh Dolatian, Mostafa Qorbani, Amir Abbas Vaezi

**Affiliations:** 1 Midwifery and Reproductive Health Research Center, Department of midwifery and Reproductive Health, School of Nursing and Midwifery, Shahid Beheshti University of Medical Sciences, Tehran, Iran; 2 Non-communicable Diseases Research Center, Alborz University of Medical Sciences, Karaj, Iran; 3 Chronic Diseases Research Center, Endocrinology and Metabolism Population Sciences Institute, Tehran University of Medical Sciences, Tehran, Iran; 4 Department of Internal Medicine, School of Medicine, Alborz University of Medical Sciences, Karaj, Iran

**Keywords:** Vitamin D, Omega-3 fatty acids, Docosahexaenoic acid, Eicosapentaenoic acid, Insulin resistance, Prediabetic state, Type 2 diabetes mellitus

## Abstract

**Background:**

A prediabetic state is a risk factor for type 2 diabetes. There are no approved drugs to manage prediabetes. Among the supplements routinely used by individuals, vitamin D and omega-3 have been studied to reduce fasting blood sugar. However, their co-supplementation has not been studied in individuals with prediabetes. This randomized controlled trial is designed to determine the effects of these two supplements on fasting blood sugar, other cardio metabolic risk factors, and psychological distress in reproductive-aged women with prediabetes and hypovitaminosis D.

**Methods/design:**

This 2 × 2 factorial, triple-blind, randomized, placebo-controlled, clinical trial will be done on 168 women of reproductive age diagnosed with prediabetes and hypovitaminosis D. Participants will be randomly assigned equally to four groups: (1) 1000 mg omega-3 fatty acid twice a day + vitamin D placebo every two weeks; (2) omega-3 fatty acid placebo twice a day + 50,000 IU vitamin D every two weeks; (3) 1000 mg omega-3 fatty acid twice a day + 50,000 IU vitamin D every two weeks; (4) omega-3 fatty acid placebo twice a day + vitamin D placebo every two weeks for eight weeks. At the beginning, participants will provide a self-reported questionnaire on the sociodemographic characteristics. At baseline and post-intervention visits, physical activity, Depression Anxiety Stress Scale 21 and Pittsburgh Sleep Quality Index, and a three-day food record will be collected for each individual. Blood pressure, weight, height, and waist circumference will also be measured.

At the beginning and at the end, a blood sample will be used for estimating serum glucose indices (fasting blood sugar and insulin, homeostasis model assessment-insulin resistance, homeostasis model assessment-beta cell function), lipids (triglyceride, low-density lipoprotein cholesterol, high-density lipoprotein cholesterol, total cholesterol), and vitamin D status.

Data analysis using Kolmogorov–Smirnov test, paired t-test, one-way analysis of variance, and repeated measures analysis of variance will be conducted through SPSS-24 software.

**Discussion:**

The primary aim of the present trial is to determine the effect of vitamin D and/or omega-3 on glycemic indices, lipid profiles, psychological distress, blood pressure, and anthropometric indices in prediabetic women with hypovitaminosis D. The results from this trial will provide evidence on the efficacy of these two supplements for preventing or delaying the onset of type 2 diabetes in high-risk individuals.

**Trial registration:**

Iran Clinical Trials Registry, IRCT20100130003226N17. Registered on 9 February 2019.

## Background

Diabetes mellitus (DM) is a common chronic disease. There has been a threefold increase in the prevalence of diabetes between 1980 and 2014 [1]. In 2017, prevalence of diabetes has been reported as 8.51% in the world and 9.6% in Iran [2]. Control of risk factors is one of the suggestions for the prevention of non-communicable diseases [3]. Prediabetes is a risk factor of diabetes mellitus and is typically defined as having either impaired glucose tolerance (IGT) or impaired fasting glucose (IFG) or both [4]. According to the International Diabetes Federation, the number of adults with prediabetes is expected to increase worldwide, to 471 million by 2035. Each year, about 5%–10% of individuals with prediabetes will progress to diabetes [5]. Prediabetes may have adverse effects on end organs, such as blood vessels, the heart, eyes, and kidneys [6].

Reproductive health in women is affected by insulin resistance. It causes depressive and fertility disorders, urinary incontinence, vaginal and urinary tract infections, menstrual changes, and sexual dysfunction and has also been linked to an increased risk of cancer of the breast and ovaries [7].

In addition, evidence suggests that psychological distress, including depression, anxiety, or stressful experiences, might affect diabetes, in terms of both its onset and its exacerbation [8]. Moreover, studies have shown that both decreased quantity and quality of sleep are associated with diabetes [9].

On the other hand, several studies have found that weight loss and increase in physical activity improved sensitivity to insulin [10, 11]. However, maintaining weight and physical activity over long periods of time is very difficult. Therefore, they are inadequate to reduce the risk of progression to diabetes [12].

As prediabetes is not known as a disease, there are currently no approved drugs to manage it.

Considering the above reasons, it is necessary to find safe and effective interventions to control prediabetes [13]. Keeping in mind that one of the suggested pathological mechanisms in the development of diabetes is subclinical inflammation, it seems that anti-inflammatory agents such as omega-3 may delay the risk of the development of diabetes in people with prediabetes [14–16].

Moreover, the results of several studies show an association of vitamin D deficiency with insulin resistance, impaired insulin secretion, and their important metabolic consequences [17–19]. In addition, a meta-analysis has declared that depression is associated with a lower level of vitamin D [20]. Further, effectiveness of omega-3 on depression has been shown in another meta-analysis [21].

In some studies, it could be seen that combined high-dose omega-3 fatty acids and high-dose vitamin D therapy improved glycemic indices in patients with new onset type 1 diabetes mellitus and women with gestational diabetes (GDM) [22, 23]. Since the effect of co-supplementation of vitamin D and omega-3 on fasting blood sugar and other cardiometabolic risk factors are not well documented, this study has been designed to evaluate the complementary or synergistic effects of vitamin D and omega-3 on fasting blood sugar and other glycemic indices, serum lipids, and psychological distress in reproductive-aged women with prediabetes and hypovitaminosis D.

## Methods/design

### Study design

This is an eight-week 2 × 2 factorial, triple-blinded (study investigators, participants, and statistical analyzer), randomized, placebo-controlled trial. Overall, 168 women of reproductive age diagnosed with prediabetes (fasting glucose 100–125 mg/mL) will be screened for enrolment in the study. This study will be conducted at Shahid Rastravesh Health Center Laboratory, Alborz University of Medical Sciences, Karaj, Iran.

Eligible participants who meet the inclusion and exclusion criteria will be randomly divided into four groups to receive double placebo (omega-3 and vitamin D placebos), omega-3 placebo and vitamin D supplement, omega-3 supplement and vitamin D placebo, and supplements of omega-3 and vitamin D.

In this factorial 2 × 2 design, we want to address five questions:

1. Compared to placebo, is omega-3 effective?
2. Compared to placebo, is vitamin D effective?
3. Are vitamin D + omega-3 more effective than placebo?
4. Are vitamin D + omega-3 more effective than vitamin D alone?
5. Are vitamin D + omega-3 more effective than omega-3 alone [24]?

**Fig. 1:**
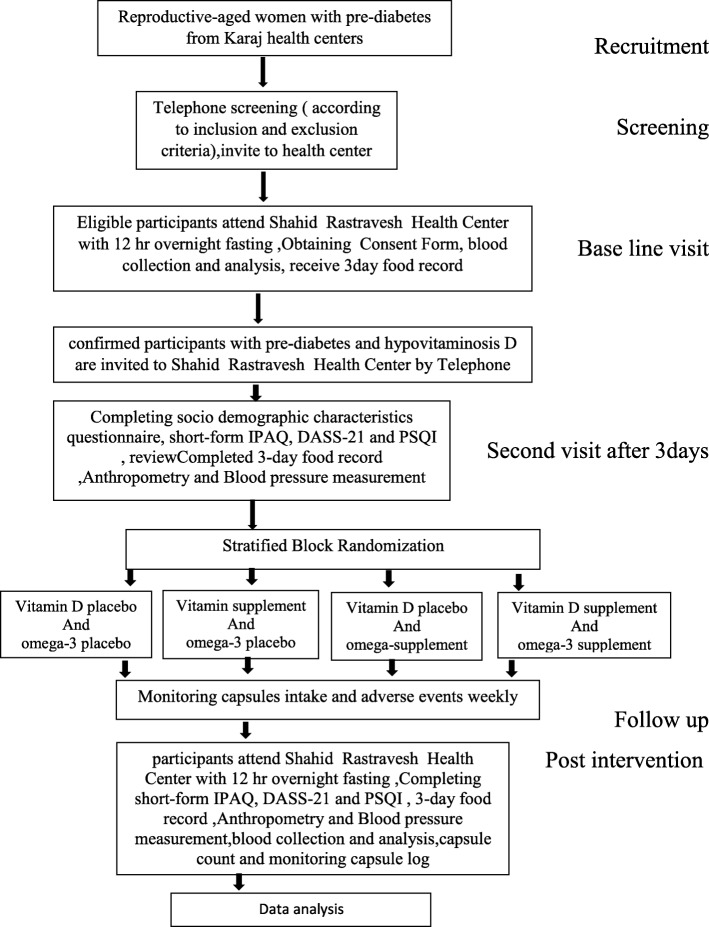
Trial protocol *flow chart*

Participants will visit the trial sites three times: at baseline (0 weeks); after three days; and post intervention (eight weeks after intervention). The flow chart of the study protocol is presented in Fig. 1.

### Objectives

1. Compare the mean serum lipid (triglycerides [TG], total cholesterol [TC], low-density lipoprotein cholesterol [LDL-C], high-density lipoprotein cholesterol [HDL-C]) and glucose indices (fasting blood sugar [FBS], fasting blood insulin [FBI], homeostasis model assessment - insulin resistance [HOMA-IR], homeostasis model assessment - beta cell function [HOMA-B]) between the four groups and within each group, before and after intervention.
2. Compare the means of anthropometric indices (weight, waist circumference, body mass index [BMI]) and systolic and diastolic blood pressures between the four groups and within each group, before and after the intervention.
3. Compare the mean scores of the Depression Anxiety Stress Scale (DASS-21) and Pittsburgh Sleep Quality Index (PSQI) between the four groups and within each group, before and after intervention.
4. Determine the relationship between changes in mean level of serum vitamin D and each of the glucose indices, serum lipids, and the mean scores of the DASS-21 and PSQI between the four groups and within each group, during intervention.

### Inclusion criteria

- Women of reproductive age (aged 15–50 years);
- FBS 100–125 mg/mL [1, 2, 25];
- Vitamin D < 32 ng/mL [26];
- BMI < 30 kg/m^2^.

### Exclusion criteria

- Diagnosed pathological conditions such as thyroid or parathyroid disorders, PCO, seizures, liver or kidney disease, neurological disorders, cancer, cardiovascular disease, type 1 or 2 diabetes, sarcoidosis, or other granulomatous disorder;
- Intake of vitamin D or omega-3 during the last six months;
- Breastfeeding, pregnant, and/or planning for pregnancy in the next two months;
- Intake of drugs that interact with omega-3 (including aspirin or anticoagulants [warfarin, heparin]) or with vitamin D (such as cardiac glycosides, cholestyramine, anticonvulsant drugs or thiazides);
- Taking herbal or chemical drugs that affect serum lipids or blood glucose level;
- In case of taking prescribed supplements different from study protocol.

### Study participants

Women of reproductive age with prediabetes from Karaj health centers diagnosed with FBS test (fasting glucose 100–125 mg/mL) [1, 25] will be invited to this study.

The study investigator will call participants by phone and will screen them according to the inclusion and exclusion criteria.

Eligible participants will be referred to the Shahid Rastravesh Health Center. First, the goals, methods, and benefits of the intervention will be explained by the investigator and the informed consent form will be signed by participants. At the beginning, 10 mL of blood will be taken from a peripheral vein after a 12-h overnight fast to assess plasma glucose levels, insulin, TG, HDL-C, TC, and vitamin D. After centrifuging for 10 min (3000 rpm), the serum samples will be frozen simultaneously and stored at − 80 °C until analyzed [12]. LDL-C will be estimated based on the Friedewald equation [27]. Participants will receive a three-day food record. The investigator will explain how to complete the form to each participant.

After three days, women who have been confirmed as having prediabetes (fasting glucose 100–125 mg/mL) and hypovitaminosis D (vitamin D < 32 ng/mL) [26] will be invited to the Shahid Rastravesh Health Center.

The sociodemographic characteristics questionnaire, short-form International Physical Activity Questionnaire (IPAQ), DASS-21, and PSQI will be completed with interviews. The investigator will review completed three-day food record in presence of each participant.

Anthropometry including weight, height, and waist circumference will be measured using a digital scale, stadiometer, and non-elastic tape, respectively.

Blood pressure (systolic and diastolic) will also be measured with a digital manometer.

All the above-mentioned stages, including blood sampling, completing four questionnaires (three-day food record, IPAQ, DASS-21, and PSQI), anthropometric measurements and measuring blood pressure, will be repeated after eight weeks.

**Fig. 2:**
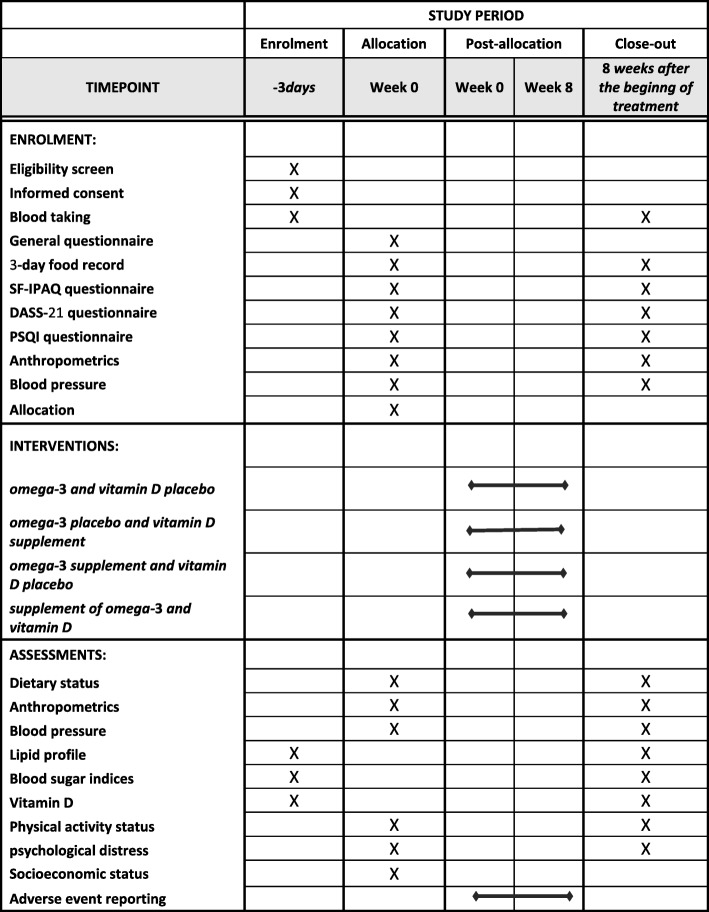
Contents of the schedule of enrolment, interventions, and assessments

At the end, the evaluated outcomes will be presented to participants privately. A Data Monitoring Committee (DMC) will also monitor during and at the end of this study (Fig. 2).

### Sample size

Sample size was estimated according to previous study using the following formula [22]:

$$ n=\frac{{\left(\kern0.28em {Z}_{1-a/2}+{Z}_{1-\beta}\kern0.28em \right)}^2\kern0.28em \left(\kern0.28em {S}_1^2+{S}_2^2\kern0.28em \right)}{{\left({\mu}_1-{\mu}_2\right)}^2} $$

Based on a study by Jamilian et al. [22], the mean ± standard deviation [SD]) of FBS in co-supplementation of vitamin D and omega-3 and placebo groups were considered as 86.8 ± 6.40 and 94.6 ± 10.30, respectively. Given the type I and II error by 5% and 20%, respectively, the calculated sample size was 20 in each group. Since we want to perform five pairwise comparisons between the study groups, the sample size was increased to 38 individuals in each group for multiple adjustment. By considering four groups and an attrition rate of 10%, the estimated sample size was 168 individuals [24].

To achieve the target sample size, all invited patients who will come the Shahid Rastravesh Health Center will be included if they are willing to participate in the study (considering the inclusion criteria of the study).

Finally, a total number of 168 women will be allocated equally to four groups using the stratified permuted block randomization method.

### Randomization method and intervention process

Eligible participants will be randomly divided into treatments with either double placebo, vitamin D alone, omega-3 alone, or double active (vitamin D and omega-3) groups according to the stratified block randomization. Block randomization is done in equal block sizes of four to ensure balance between groups. Stratified randomization will be used to control serum vitamin D level (vitamin D < 20 ng/mL, range = 20–32 ng/mL) [28] distribution. Any individual who is not involved in trial data collection and analysis carries out randomization. The intervention allocation will be blinded to the study investigators, participants, and the statistical analyzer.

Zahravi Pharmaceutical Company (Tabriz, Iran) produced vitamin D and omega-3 supplements and placebos. This was approved by the Food and Drug Administration. The appearance of the placebo capsule was indistinguishable in color, shape, size, packaging, smell, and taste from vitamin D and omega-3 capsules. The type of supplements in each group will be blinded as A, B, C, and D packages for investigators, participants, and the statistical analyzer.

Participants will receive supplements in four groups for eight weeks:

1. Vitamin D placebo every two weeks + 1000 mg omega-3 containing 360 mg eicosapentaenoic acid (EPA) and 240 mg docosahexaenoic acid (DHA) twice a day;
2. 50,000 IU vitamin D every two weeks + omega-3 placebo twice a day;
3. 50,000 IU vitamin D every two weeks + 1000 mg omega-3 twice a day;
4. Vitamin D placebo every two weeks + omega-3 placebo twice a day.

At the beginning of the study, participants will receive the supplements. Previous studies have not reported any side effects from the supplements [22, 23]. Compliance of the participant to the intervention will be measured using capsule count and capsule log record. Their potential complications and consumption processes will be registered (number of consumed capsules and the returned packages). In addition, the consumption process will be checked once a week by telephone and any occurrence of adverse events will be recorded. Participants will be asked to keep their usual lifestyle including nutrition and physical activity level (Additional file 1).

### Study outcomes

#### Primary outcomes

The primary outcome of this clinical trial is the difference in FBS in eight weeks between double placebo, vitamin D, omega-3, and double active groups from baseline to the post-intervention visit.

#### Secondary outcomes

The secondary outcomes of this clinical trial are changes in other glycemic indices, lipid profiles, psychological distress, anthropometric indices, and blood pressure at the end of the study in comparison with the baseline values.

### Assessments and measurements

Participants will be visited at the Shahid Rastravesh Health Center three times, at baseline, after three days and after eight weeks. Participants will be interviewed regarding their sociodemographic background at baseline.

Then, at the beginning and eight weeks after the intervention, the following will be done:

- Blood sample will be drawn after at least 12 h of fasting. All blood samples will be centrifuged at 3000 rpm for 10 min and serum will be separated into clean tube aliquots and stored at – 80 °C until analysis [12]. Collection of blood, specimen storage, and laboratory tests will be conducted at the Shahid Rastravesh Health Center laboratory in Karaj. To determine FBS, serum triglycerides, TC, and HDL-C concentrations, we will use an enzymatic kit (Pars Azmun, Tehran, Iran). LDL-C will be estimated based on the Friedewald equation [27]. Serum level of vitamin D and insulin will be quantified using a commercial enzyme-linked immunosorbent assay kit (Monobind, CA, USA).
  The HOMA-IR and HOMA-B will be determined according to suggested formulas [29].

$$ \mathrm{HOMA}-\mathrm{IR}=\left[\mathrm{FBI}\ \left(\upmu \mathrm{U}/\mathrm{mL}\right)\mathrm{x}\ \mathrm{FBS}\ \left(\mathrm{mg}/\mathrm{ml}\right)\right]/405 $$

$$ \mathrm{HOMA}-\mathrm{B}=\left[\mathrm{FBI}\ \left(\upmu \mathrm{U}/\mathrm{mL}\right)\ \mathrm{x}\ 360/\mathrm{FBS}\ \left(\mathrm{mg}/\mathrm{ml}\right)-63\right]\% $$

- Weight, height, and waist circumference will be determined using a digital scale, stadiometer, and non-elastic tape, respectively. They are measured thus: weight without shoes, with minimal clothing, and with a 100-g accuracy (Beurer, BF220, Germany); height without shoes, standing, heels against the wall, flat and forward head, and with 0.5-cm accuracy (Seca, Hamburg, Germany); and waist circumference with minimal clothing, midway between the last rib and the iliac crest. BMI will be calculated using the height and weight measurements kg/m^2^ [12].
- Systolic and diastolic blood pressure values will be measured with a digital manometer (Beurer, Germany). Values will be recorded in mmHg [30] .
- Physical activity will be investigated the short form of IPAQ [31]. The IPAQ form comprises walking, moderate intensity, and vigorous intensity activity and will be expressed as metabolic equivalents per minute (MET min) per week. The levels of physical activity will be categorized into low, moderate, and high based on the IPAQ criteria. This questionnaire has been validated in previous studies [32] including in Iran [33].
- Food intake status will be assessed using the three-day food records (comprising two working days and one weekend). Modified Nutritionist-4 software program (First Databank, San Bruno, CA, USA) will be used to estimate the energy and nutrient intakes.
- DASS-21 and PSQI will be applied to assess psychological distress [34, 35].
- DASS was designed to measure emotional distress in three subcategories [34] of depression (e.g. loss of self-esteem/incentives and depressed mood), anxiety (e.g. fear and anticipation of negative events), and stress (e.g. persistent state of over arousal and low frustration tolerance).
  DASS is a self-report scale with 21 items (seven items for each category) based on a 4-point rating scale. To calculate comparable scores with full DASS, each seven-item scale will be multiplied by two. Participants will be asked to explain times of occurrence of each item (in the form of statements) over the past week, with “0 = did not apply to me at all” to “3 = applied to me very much, or most of the time.” The higher score shows more severe emotional distress. Validity and reliability of the DASS-42 were examined in several studies and in Iranian samples [36–38].
- The PSQI is a self-report questionnaire that assesses sleep quality and quantity [35]. The19-item self-report questionnaire yields seven component scores: subjective sleep quality; sleep latency; sleep duration; habitual sleep efficiency; sleep disturbances; use of sleeping medication; and daytime disfunction.
- There are five additional questions. These are not used in the scoring. A Global Sleep Quality score > 5 indicates poor sleep quality. There is evidence of the reliability and validity of the PSQI in several subjects [39–42] and also in Iran [43, 44].

### Statistical analysis

The effect of the intervention on all participants will be assessed based on intention-to-treat and per-protocol analyses. The data will be checked for plausibility by randomly checking the accuracy and completeness and source data verification. The normality of continuous variables will be determined using a Kolmogorov–Smirnov test. Appropriate statistical transformation (logarithmic transformation, square root transformation, cube root transformation, or reciprocal transformation) will be used for continuous variables with not normal distribution.

Hence, statistical analysis for continuous variables with normal distribution (parametric tests) will be used in this study.

Continuous variables with normal distribution and transformed variables will be expressed as mean ± SD. Categorical data will be presented as frequency (percentage).

To compare the mean differences within each group, before and after intervention, the paired t-test will be used for continuous variables.

For continuous variables, one-way analysis of variance (ANOVA) will be used to detect differences in sociodemographic characteristics, metabolic profiles, physical activity levels, and dietary intakes at the study baseline between the four groups.

Categorical data at the study baseline will be analyzed using the Chi-squared test between the intervention groups.

Repeated measures analysis of variance (RM ANOVA) with Bonferroni procedure to adjust for the five comparisons will be used to assess time effects and time-by-treatment (vitamin D and/or omega-3) interaction effects on all outcome variables between groups.

Since we have multiple endpoints and we will compare five pairwise comparison, all *p* values will be corrected using the Bonferroni adjustment method.

Two-way RM ANOVA with Bonferroni adjustment for comparisons will be used to assess the relationship between changes in mean serum vitamin D level and each of fasting blood sugar and other outcome variables between groups.

Levenes test for independent data in one-way ANOVA and Mauchly’s test in RM ANOVA will be used to test homoscedasticity.

All statistical measurements will be reported with 95% confidence intervals (CI). A *p* value < 0.05 will be considered statistically significant. All statistical analyses will be performed using SPSS version 24.

## Discussion

Diabetes has become one of the leading public health issues in the world and its prevention is a major objective of the World Health Organization (WHO) [2]. Prediabetes is a risk factor for type 2 diabetes. There is therefore a necessity for safe and cost-effective agents that can improve insulin sensitivity and beta-cell function in high-risk individuals [13].

On the other hand, vitamin D deficiency has become a common problem globally. Almost one billion people worldwide are estimated to live with vitamin D deficiency [45].

Low vitamin D levels are associated with higher fasting glucose [18, 46]. This association is found in both cross-sectional studies and prospective population studies [47–49].

Although conflicts in the results of previous supplementation studies are seen [4], there are several mechanisms that vitamin D might alter glucose metabolism. Vitamin D has anti-inflammatory and immune modulatory effects [50] and may also stimulate insulin release by pancreatic b-cells [51, 52].

There is a hypothesis that omega-3 supplementation may improve glycemic control, although the its mechanisms are not clear yet [53]. During a study on animal models, the following potential mechanisms were found: hepatic insulin sensitivity improvement [54] by hepatic fatty acid oxidation and reducing lipogenesis [55, 56]; increased production of adipocytokines (e.g. adiponectin and leptin) [57]; direct [58] and indirect [55] anti-inflammatory effects and associated improvements in insulin sensitivity in the liver, muscle, and adipose tissue; and modulation of incretin hormones, which are involved in glucose-stimulated insulin secretion [59].

Lack of n-3 PUFA effects on insulin sensitivity has been found in a meta-analysis [60], while association of short-term fish oil supplementation with increasing insulin sensitivity has been shown in another meta-analysis among people with metabolic disorders [61]. However, two studies have shown improved glycemic status in patients with new onset type 1 diabetes mellitus and women with GDM with combined high-dose omega-3 and high-dose vitamin D therapy [22, 23]. Considering the results of the two studies mentioned above, the combination of vitamin D and omega-3 might provide either synergistic or complementary effects in prediabetes, which will be evaluated through this 2 × 2 factorial study design. Vitamin D interfering with omega-3 was not reported in previous studies.

This is the first randomized controlled trial that will determine the effect of vitamin D and omega-3 on glycemic status, serum lipids, and psychological distress in women of reproductive age with prediabetes and hypovitaminosis D. This trial, therefore, investigates potential effects of these two supplements in decreasing the risk factors of type 2 diabetes.

### Strengths and limitation of study design

The strengths of this study are its factorial design to assess the individual, complementary or synergistic effects of both interventions, and triple blinding of the participants, investigators, and statistical analyzer to reduce bias.

The advice to participants is to maintain a steady pattern of diet and physical activity. Reducing the impact of confounding factors, two questionnaires evaluate their patterns.

To assess compliance with the intervention, a capsule log and a capsule count are used.

In previous studies, toxic or adverse effect at this dose of vitamin D and omega-3 have not been reported. In this study, all of the risk factors are not evaluated so the results may not be enough to reduce the risk of diabetes completely.

### Trial status

This article refers to the protocol version 3 dated 10 June 2019. The recruitment will begin on 2 February 2019, with completion of recruitment expected in September 2019.

## Supplementary information

**Additional file 1.** SPIRIT 2013 Checklist: Recommended items to address in a clinical trial protocol and related documents*.

## Data Availability

The data will not be shared because it is a protocol and results are secure before publishing.

## Abbreviations

BMI: Body mass index
DASS-21: Depression Anxiety Stress Scale
DM: Diabetes mellitus
FBI: Fasting blood insulin
FBS: Fasting blood sugar
HDL-C: High-density lipoprotein cholesterol
HOMA-B: Homeostatic model assessment for B-cell function-B)
HOMA-IR: Homeostasis model assessment estimated insulin resistance
IPAQ: International Physical Activity Questionnaire
LDL: Low-density lipoprotein
PSQI: Pittsburgh Sleep Quality Index
T2DM: Type 2 diabetes mellitus
TC: Total cholesterol
TG: Triglyceride

## References

[1] World Health Organization (2016). Global Report on Diabetes.

[2] World Bank (2017). Diabetes prevalence.

[3] National Action Plan for Prevention and Control of Non-Communicable Diseases and the Related Risk Factors in the Islamic Republic of Iran, 2015–2025. Iranian National Committee for NCDs Prevention and Control; 2015.

[4] George PS, Pearson ER, Witham MD (2012). Effect of vitamin D supplementation on glycaemic control and insulin resistance: a systematic review and meta-analysis. Diabet Med.

[5] International Diabetes Federation Diabetic Atlas. 2017. https://www.idf.org/elibrary/epidemiology-research/diabetes-atlas.html. Accessed 10 Jan 2019.

[6] Tabák AG, Herder C, Rathmann W, Brunner EJ, Kivimäki M (2012). Prediabetes: a high-risk state for diabetes development. Lancet.

[7] Rezai S, LoBue S, Henderson CE (2016). Diabetes prevention: Reproductive age women affected by insulin resistance. Womens Health.

[8] Farr SL, Hayes DK, Bitsko RH, Bansil P, Dietz PM (2011). Depression, diabetes, and chronic disease risk factors among US women of reproductive age. Prev Chronic Dis.

[9] Cespedes EM, Dudley KA, Sotres-Alvarez D, Zee PC, Daviglus ML, Shah NA (2016). Joint associations of insomnia and sleep duration with prevalent diabetes: The Hispanic Community Health Study/Study of Latinos (HCHS/SOL). J Diabetes.

[10] Defronzo RA, Abdul-Ghani MA (2011). Preservation of β-cell function: the key to diabetes prevention. J Clin Endocrinol Metab.

[11] Nathan DM, Davidson MB, DeFronzo RA, Heine RJ, Henry RR, Pratley R (2007). Impaired fasting glucose and impaired glucose tolerance: implications for care. Diabetes Care.

[12] Aghasi M, Ghazi-Zahedi S, Koohdani F, Siassi F, Nasli-Esfahani E, Keshavarz A (2018). The effects of green cardamom supplementation on blood glucose, lipids profile, oxidative stress, sirtuin-1 and irisin in type 2 diabetic patients: a study protocol for a randomized placebo-controlled clinical trial. BMC Complement Altern Med.

[13] Thota RN, Acharya SH, Abbott KA, Garg ML (2016). Curcumin and long-chain Omega-3 polyunsaturated fatty acids for Prevention of type 2 Diabetes (COP-D): study protocol for a randomised controlled trial. Trials..

[14] Bahadoran Z, Mirmiran P, Azizi F (2013). Dietary polyphenols as potential nutraceuticals in management of diabetes: a review. J Diabetes Metab Disord.

[15] Hommelberg PP, Langen RC, Schols AM, Mensink RP, Plat J (2010). Inflammatory signaling in skeletal muscle insulin resistance: green signal for nutritional intervention?. Curr Opin Clin Nutr Metab Care.

[16] Li D (2015). Omega-3 polyunsaturated fatty acids and non-communicable diseases: Meta- analysis based systematic review. Asia Pac J Clin Nutr.

[17] Grundy SM (2002). Third report of the national cholesterol education program (NCEP) expert panel on detection, evaluation, and treatment of high blood cholesterol in adults (Adult Treatment Panel III) final report. Circulation..

[18] Hyppönen E, Power C (2006). Vitamin D status and glucose homeostasis in the 1958 British birth cohort: the role of obesity. Diabetes Care.

[19] Pittas AG, Dawson-Hughes B (2010). Vitamin D and diabetes. J Steroid Biochem Mol Biol.

[20] Anglin RE, Samaan Z, Walter SD, McDonald SD (2013). Vitamin D deficiency and depression in adults: systematic review and meta-analysis. Br J Psychiatry.

[21] Martins JG (2009). EPA but not DHA appears to be responsible for the efficacy of omega-3 long chain polyunsaturated fatty acid supplementation in depression: evidence from a meta-analysis of randomized controlled trials. J Am Coll Nutr.

[22] Jamilian M, Samimi M, Ebrahimi FA, Hashemi T, Taghizadeh M, Razavi M (2017). The effects of vitamin D and omega-3 fatty acid co-supplementation on glycemic control and lipid concentrations in patients with gestational diabetes. J Clin Lipidol.

[23] Baidal DA, Ricordi C, Garcia-Contreras M, Sonnino A, Fabbri A (2016). Combination high-dose omega-3 fatty acids and high-dose cholecalciferol in new onset type 1 diabetes: a potential role in preservation of beta-cell mass. Eur Rev Med Pharmacol Sci.

[24] Freidlin B, Korn EL (2017). Two-by-Two Factorial Cancer Treatment Trials: Is Sufficient Attention Being Paid to Possible Interactions?. J Natl Cancer Inst.

[25] Diabetes Care. 2013;36(Suppl 1):S.67–74.

[26] Spedding Simon, Vanlint Simon, Morris Howard, Scragg Robert (2013). Does Vitamin D Sufficiency Equate to a Single Serum 25-Hydroxyvitamin D Level or Are Different Levels Required for Non-Skeletal Diseases?. Nutrients.

[27] Płudowski Paweł, Karczmarewicz Elżbieta, Bayer Milan, Carter Graham, Chlebna-Sokół Danuta, Czech-Kowalska Justyna, Dębski Romuald, Decsi Tamas, Dobrzańska Anna, Franek Edward, Głuszko Piotr, Grant William B., Holick Michael F., Yankovskaya Liudmila, Konstantynowicz Jerzy, Książyk Janusz B., Księżopolska-Orłowska Krystyna, Lewiński Andrzej, Litwin Mieczysław, Lohner Szimonetta, Lorenc Roman S., Łukaszkiewicz Jacek, Marcinowska-Suchowierska Ewa, Milewicz Andrzej, Misiorowski Waldemar, Nowicki Michał, Povoroznyuk Vladyslav, Rozentryt Piotr, Rudenka Ema, Shoenfeld Yehuda, Socha Piotr, Solnica Bogdan, Szalecki Mieczysław, Tałałaj Marek, Varbiro Szabolcs, Żmijewski Michał A. (2013). Practical guidelines for the supplementation of vitamin D and the treatment of deficits in Central Europe — recommended vitamin D intakes in the general population and groups at risk of vitamin D deficiency. Endokrynologia Polska.

[28] Friedewald WT, Levy RI, Fredrickson DS (1972). Estimation of the concentration of low-density lipoprotein cholesterol in plasma, without use of the preparative ultracentrifuge. Clin Chem.

[29] Pisprasert V, Ingram KH, Lopez-Davila MF, Munoz AJ, Garvey WT (2013). Limitations in the use of indices using glucose and insulin levels to predict insulin sensitivity: impact of race and gender and superiority of the indices derived from oral glucose tolerance test in African Americans. Diabetes Care.

[30] Javidi A, Mozaffari-Khosravi H, Nadjarzadeh A, Dehghani A, Eftekhari MH (2016). The effect of flaxseed powder on insulin resistance indices and blood pressure in prediabetic individuals: A randomized controlled clinical trial. J Res Med Sci.

[31] Ainsworth BE, Haskell WL, Whitt MC, Irwin ML, Swartz AM, Strath SJ (2000). Compendium of Physical Activities: an update of activity codes and MET intensities. Med Sci Sports Exerc.

[32] Craig CL, Marshall AL, Sjorstrom M, Bauman AE, Booth ML, Ainsworth BE (2003). International physical activity questionnaire: 12-country reliability and validity. Med Sci Sports Exerc.

[33] Hazavehei S, Asadi Z, Hasanzade A, Shekarchizadeh P (2009). A study on the effect of physical education (Π) curriculum based on BASNEF model on female students’ regular physical activity in Isfahan University of Medical Sciences. ZUMS Journal.

[34] Lovibond S, Lovibond P (1995). Manual for the depression anxiety stress scales.

[35] Buysse DJ, Reynolds CF, Monk TH, Berman SR, Kupfer DJ (1989). The Pittsburgh Sleep Quality Index: a new instrument for psychiatric practice and research. Psychiatry Res.

[36] Sahebi A, Asghari MJ, Salari RS (2005). Validation of Depression Anxiety and Stress Scale (DASS-21) for an Iranian population. J Iran Psychol.

[37] Moradipanah F, Mohammadi E, Mohammadil A (2009). Effect of music on anxiety, stress, and depression levels in patients undergoing coronary angiography. East Mediterr Health J.

[38] Samani S, Joukar B (2007). A study on the reliability and validity of the short form of the depression anxiety stress scale (dass-21). J Soc Sci Hum Shiraz Univ.

[39] Gentili A, Werner DK, Kuchibhatla M, Edinger JD (1995). Test-retest reliability of the Pittsburgh sleep quality index in nursing home residents. J Am Geriatr Soc.

[40] Reynolds CF, Hoch CC, Buysse DJ, Houck PR, Schlernitzauer M, Pasternak RE (1993). Sleep after spousal bereavement: a study of recovery from stress. Biol Psychiatry.

[41] Rubinstein ML, Selwyn PA (1998). High prevalence of insomnia in an outpatient population with HIV infection. J Acquir Immune Defic Syndr Hum Retrovirol.

[42] Stein MB, Chartier M, Walker JR (1993). Sleep in nondepressed patients with panic disorder: I. Systematic assessment of subjective sleep quality and sleep disturbance. Sleep..

[43] Afkham Ebrahimi A, Bandi G, Salehi M, Tafti K, Vakili Y, Farsi A (2008). Sleep parameters and the factors affecting the quality of sleep in patients attending selected clinics of Rasoul-e-Akram hospital. Razi Journal of Medical Sciences..

[44] Malek M, Halvani G, Fallah H, Jafari Nodoushan R (2011). A study of the relationship between the Pittsburgh sleep quality index and road accidents among truck drivers. Occupational Medical Journal.

[45] Holick MF (2007). Vitamin D deficiency. N Engl J Med.

[46] Kositsawat J, Freeman VL, Gerber BS, Geraci S (2010). Association of A1C levels with vitamin D status in US adults: data from the National Health and Nutrition Examination Survey. Diabetes Care.

[47] Hyppönen E, Boucher BJ, Berry DJ, Power C (2008). 25-hydroxyvitamin D, IGF-1, and metabolic syndrome at 45 years of age: a cross-sectional study in the 1958 British Birth Cohort. Diabetes..

[48] Knekt Paul, Laaksonen Maarit, Mattila Catharina, Härkänen Tommi, Marniemi Jukka, Heliövaara Markku, Rissanen Harri, Montonen Jukka, Reunanen Antti (2008). Serum Vitamin D and Subsequent Occurrence of Type 2 Diabetes. Epidemiology.

[49] Grimnes G, Emaus N, Joakimsen RM, Figenschau Y, Jenssen T, Njølstad I (2010). Baseline serum 25-hydroxyvitamin D concentrations in the Tromsø Study 1994–95 and risk of developing type 2 diabetes mellitus during 11 years of follow-up. Diabet Med.

[50] Hewison M (2010). Vitamin D and the immune system: new perspectives on an old theme. Endocrinol Metab Clin N Am.

[51] Pitocco D, Crino A, Di Stasio E, Manfrini S, Guglielmi C, Spera S (2006). The effects of calcitriol and nicotinamide on residual pancreatic β-cell function in patients with recent onset Type 1 diabetes (IMDIAB XI). Diabetic Med.

[52] Borissova A, Tankova T, Kirilov G, Dakovska L, Kovacheva R (2003). The effect of vitamin D3 on insulin secretion and peripheral insulin sensitivity in type 2 diabetic patients. Int J Clin Pract.

[53] Lalia A, Lanza I (2016). Insulin-sensitizing effects of omega-3 fatty acids: Lost in translation?. Nutrients.

[54] Matsuura B, Kanno S, Minami H, Tsubouchi E, Iwai M, Matsui H (2004). Effects of antihyperlipidemic agents on hepatic insulin sensitivity in perfused Goto-Kakizaki rat liver. J Gastroenterol.

[55] Wu JH, Micha R, Imamura F, Pan A, Biggs ML, Ajaz O (2012). Omega-3 fatty acids and incident type 2 diabetes: a systematic review and meta-analysis. Br J Nutr.

[56] Kuda O, Jelenik T, Jilkova Z, Flachs P, Rossmeisl M, Hensler M (2009). n-3 fatty acids and rosiglitazone improve insulin sensitivity through additive stimulatory effects on muscle glycogen synthesis in mice fed a high-fat diet. Diabetologia..

[57] Rossi AS, Lombardo YB, Lacorte J-M, Chicco AG, Rouault C, Slama G (2005). Dietary fish oil positively regulates plasma leptin and adiponectin levels in sucrose-fed, insulin-resistant rats. Am J Physiol Regul Integr Comp Physiol.

[58] Oh DY, Talukdar S, Bae EJ, Imamura T, Morinaga H, Fan W (2010). GPR120 is an omega-3 fatty acid receptor mediating potent anti-inflammatory and insulin-sensitizing effects. Cell..

[59] Flachs P, Rossmeisl M, Kopecky J (2014). The effect of n-3 fatty acids on glucose homeostasis and insulin sensitivity. Physiol Res.

[60] Akinkuolie AO, Ngwa JS, Meigs JB, Djousse L (2011). Omega-3 polyunsaturated fatty acid and insulin sensitivity: a meta-analysis of randomized controlled trials. Clin Nutr.

[61] Gao H, Geng T, Huangand T, Zhao Q (2017). Fish oil supplementation and insulin sensitivity: a systematic review and metaanalysis. Lipids Health Dis.

